# Local breast density at lesion sites in diagnostic and previous screening mammograms

**DOI:** 10.1186/bcr3699

**Published:** 2014-11-03

**Authors:** M Otsuka, E Harkness, X Chen, E Moschidis, M Bydder, S Gadde, Y Lim, A Maxwell, DG Evans, A Howell, P Stavrinos, M Wilson, S Astley

**Affiliations:** 1 University of Manchester Medical School, Manchester, UK; 2 Centre for Imaging Sciences, Institute of Population Health, University of Manchester, UK; 3 Nightingale Centre and Genesis Prevention Centre, University Hospital of South Manchester, Manchester, UK; 4 Manchester Breast Centre, Manchester Cancer Research Centre, University of Manchester, UK; 5 The University of Manchester, Manchester Academic Health Science Centre, University Hospital of South Manchester NHS Foundation Trust, Manchester, UK

## Introduction

High overall breast density is associated with increased risk of developing cancer. Semi-automated analysis of digitised analogue mammograms has previously suggested that local increases in density may occur prior to cancers being detected. We investigate local density at the site of cancers in diagnostic and previous full-field digital screening mammograms using quantitative measures.

## Methods

Volpara® volumetric breast density maps were obtained for 54 mammograms in which unilateral breast cancer was detected, and the corresponding previous digital screening mammograms that had been read as normal. A 5 mm square region was sampled from CC-view density maps at the lesion site in both diagnostic and previous screening mammograms, and in corresponding locations on the opposite side. Local percent density was computed.

## Results

In previous screening mammograms, local breast density was significantly increased at the future lesion site compared with a similar location in the opposite breast (medians 18.82%, 9.45%, *P *< 0.001). Breast density at the lesion site in diagnostic mammograms was higher than that of a corresponding area in the opposite breast (medians 21.58%, 9.18%, *P *< 0.001). It was also greater than the density of the same location in the previous screening mammogram (*P *= 0.012).

## Conclusion

Local breast density is increased at sites where cancer will develop in the future compared with corresponding regions in the opposite breast. Cancer sites in diagnostic images have greater density than the same region in previous screening mammograms and corresponding contralateral regions. Detection of localised increases in breast density could enhance computer-aided detection of early breast cancer.

